# Liver Grafts with Major Extended Donor Criteria May Expand the Organ Pool for Patients with Hepatocellular Carcinoma

**DOI:** 10.3390/jcm8101692

**Published:** 2019-10-15

**Authors:** Vladimir J. Lozanovski, Larissa T.B. Kerr, Elias Khajeh, Omid Ghamarnejad, Jan Pfeiffenberger, Katrin Hoffmann, De-Hua Chang, Markus Mieth, Thomas Longerich, Oliver Strobel, Karl Heinz Weiss, Markus W. Büchler, Arianeb Mehrabi

**Affiliations:** 1 Department of General, Visceral and Transplant Surgery, University Hospital Heidelberg, 69120 Heidelberg, Germany; Vladimir.Lozanovski@med.uni-heidelberg.de (V.J.L.);; 2 Liver Cancer Center Heidelberg (LCCH), University of Heidelberg, 69120 Heidelberg, Germany; 3 Department of Internal Medicine, University Hospital Heidelberg, 69120 Heidelberg, Germany; 4 Department of Radiology, University Hospital Heidelberg, 69120 Heidelberg, Germany; 5 Institute of Pathology, University Hospital Heidelberg, 69120 Heidelberg, Germany

**Keywords:** major extended donor criteria, HCC, liver transplantation

## Abstract

The major extended donor criteria (maEDC; steatosis >40%, age >65 years, and cold ischemia time >14 h) influence graft and patient outcomes after liver transplantation. Despite organ shortages, maEDC organs are often considered unsuitable for transplantation. We investigated the outcomes of maEDC organ liver transplantation in patients with hepatocellular carcinoma (HCC). Two hundred and sixty-four HCC liver transplant patients were eligible for analysis. Risk factor analysis was performed for early allograft dysfunction; primary nonfunction; 30-day and 90-day graft failure; and 30-day, 90-day, and 1-year patient mortality. One-year graft survival was higher in recipients of no-maEDC grafts. One-year patient survival did not differ between the recipients of no-maEDC and maEDC organs. The univariate and multivariate analyses revealed no association between maEDC grafts and one-year patient mortality. Graft survival differed between the recipients of no-maEDC and maEDC organs after correcting for a laboratory model of end-stage liver disease (labMELD) score with a cut-off value of 20, but patient survival did not. Patient survival did not differ between recipients who did and did not meet the Milan criteria and who received grafts with and without maEDC. Instead of being discarded, maEDC grafts may expand the organ pool for patients with HCC without impairing patient survival or recurrence-free survival.

## 1. Introduction

Hepatocellular carcinoma (HCC) is one of the most common causes of cancer-related death worldwide [1]. Surgery is the preferred treatment for HCC patients with preserved liver function, whereas liver transplantation remains the gold standard and only promising treatment for HCC patients with advanced liver disease or liver cirrhosis because it involves radical oncological resection and improves underlying liver dysfunction. However, patients with HCC have the longest waiting times for a transplant and 7–55% of them drop out of the waiting list because of disease progression [2]. The requirement for donor organs still exceeds the supply in most countries, so extended donor criteria (EDC) are used when selecting grafts for transplantation; however, recipients should be carefully selected to achieve acceptable outcomes after transplantation [3]. Biopsy-proven macrovesicular steatosis (BPS) >40%, donor age >65 years, and cold ischemia time (CIT) >14 h are the major EDC (maEDC) that influence graft and patient outcomes [3]. In addition, different combinations of maEDC and laboratory model of end-stage liver disease (labMELD) scores have different impacts on the outcome [3]. In our previous study, we showed that transplanting liver grafts with more than one maEDC into patients with labMELD scores ≥ 20 yielded worse outcomes, and we therefore suggested that the number of maEDC should be balanced with the recipient’s condition [3]. The use of maEDC grafts is unavoidable because of the chronic organ shortage in Eurotransplant, and the suboptimal quality of these grafts has worsened outcomes after liver transplantation [3]. Therefore, despite organ shortage, these grafts are often discarded because they are considered unsuitable for transplantation [4]. This study examines if patients with HCC and liver cirrhosis who are generally in a better condition are affected by maEDC grafts, and investigates the plausibility of an alternative approach to expanding the organ pool for these transplant candidates.

## 2. Methods

### 2.1. Patient Data Collection

We reviewed data from 1430 liver transplants performed at our center between January 2001 and September 2018. HCC patients were identified from a prospective database and data were extracted from a comprehensive transplant recipient registry, written and electronic medical records, and Eurotransplant records. All patients were listed for transplantation according to Eurotransplant procedures and protocols. Transplant candidates who were granted standard exceptional MELD (eMELD) received MELD points that started at a fixed initial value (equivalent of 15% of the 90-day predicted mortality, which means that a patient started with an eMELD of 22). The score was then upgraded by 10% of the eMELD every 90 days as long as the defining condition persisted. If the labMELD score was higher than the eMELD at time of matching, the labMELD was used for allocation [5]. The labMELD scores of patients who received a transplant before MELD score-based allocation was implemented were calculated retrospectively. Eurotransplant offered EDC grafts in a center-based fashion, allowing centers to choose a suitable recipient from the waiting list. All transplant candidates were offered the option of an EDC-organ transplantation before they were listed for the procedure, and written consent was obtained from all patients who opted in for EDC-liver transplantation. The assessment of Milan and the University of California San Francisco (UCSF) criteria was image-based according to current guidelines at the time of the evaluation for liver transplantation. Surgical procedures were performed using standard techniques [6]. Our institutional review board approved the study (reference number: S-195/2015).

**Figure 1:**
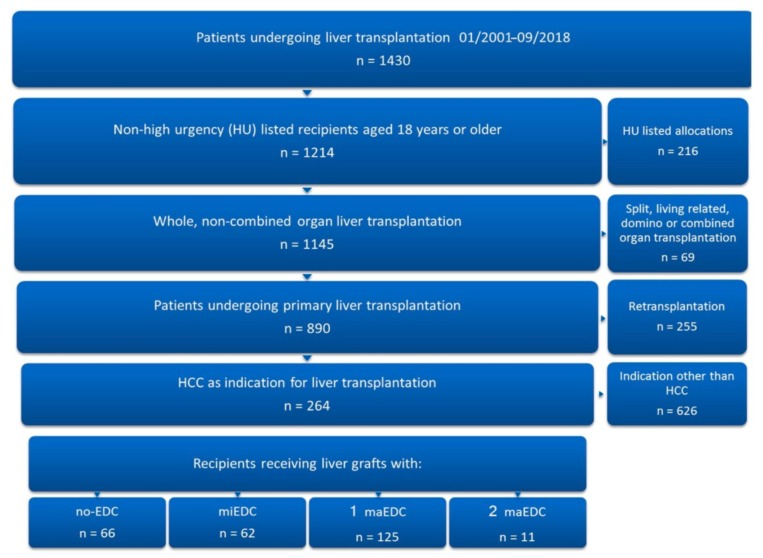
Patient selection. Of 1430 patients who underwent liver transplantation at our institution from January 2001 to September 2018, 264 were eligible for inclusion in the study.

We excluded recipients under 18 years of age, living donation transplantations, split liver transplantations, combined organ transplantations, high-urgency transplantations, and retransplantations. Two hundred and sixty-four patients were eligible for analysis. The mean donor age was 61 ± 16 years, and 58% of donors were male. The mean recipient age was 57 ± 7 years and 86% of recipients were male. The mean CIT was 10.2 ± 2.53 h (Table 1). In all cases, the indication for transplantation was HCC due to liver cirrhosis (Figure 1). The leading cause of cirrhosis was chronic hepatitis infection (52.6%) followed by chronic alcoholism (34.1%), and 8.3% of patients were diagnosed with cryptogenic liver cirrhosis (Table 1).

### 2.2. Major EDC and Assessment of Clinical Outcome

A BPS o > 40%, donor age of >65 years, and CIT of >14 h have been identified as factors that influence 3-year graft failure and patient survival after liver transplantation and were regarded as major EDC (maEDC), whereas body mass index of >30 kg/m^2^, history of previous drug abuse, history of extrahepatic malignancy, peak serum sodium (Na+) >165 mmol/L, bilirubin >3 mg/dL, alanine aminotransferase or aspartate aminotransferase greater than three times the normal level, positive hepatitis serology (HBs antigen, anti-HBc, anti-HCV positive), and duration of intensive care unit stay and/or duration mechanical ventilation >7 days prior to procurement were regarded as minor EDC (miEDC) [3,7,8,9,10]. All grafts worthy of caution prior to implantation (conspicuous macroscopic appearance of the liver, higher donor age, or history of hepatitis or alcohol consumption) were biopsied and an experienced liver pathologist at our center examined the samples. Early allograft dysfunction was defined by the presence of one or more of the following parameters: total bilirubin ≥10 mg/dL (171 μmol/L) or international normalized ratio ≥1.6 on day 7 and alanine/aspartate transaminases >2000 IU/L within the first 7 days. Primary nonfunction was defined as nonrecoverable liver function requiring retransplantation or causing death within 15 days (days 0–14) after the primary transplantation [3,11,12]. One-year graft failure was defined as insufficient liver function to keep the patient alive, leading to death or retransplantation during the first year after transplantation. Patient survival was defined as the time between the initial (primary) liver transplantation and death or last known contact. The study also aimed to examine the effect of maEDC on the post-transplant outcome. We therefore examined mortality for reasons other than graft failure-related complications (death with functioning graft), as previously reported [3]. The labMELD score was calculated to assess the recipient’s condition objectively and a cut-off value of 20 was chosen based on a previous study [11]. Risk factor analysis was performed for early allograft dysfunction; primary nonfunction; 30-day and 90-day graft failure; and 30-day, 90-day, and 1-year patient mortality. Ninety-day graft failure was defined as a short-term graft outcome. One-year patient mortality was chosen to reduce the mortality bias due to HCC recurrence, but we also analyzed the oncologic outcome and examined patient overall survival, HCC recurrence rates, and recurrence-free survival. Recurrence-free survival was defined as the time between the transplantation and the date of the first detected recurrence or the last follow-up visit without recurrence. Cox regression analyses of donor and recipient factors associated with 1-year graft failure and patient mortality were performed and recipient age, gender, body mass index >30 kg/m^2^, tumor grade, labMELD score, cause of liver cirrhosis, Milan criteria, Child–Pugh score, and maEDC were analyzed. Follow-up ended on the last documented day of contact, and the median follow-up was 68 (range 1–190) months.

### 2.3. Statistical Analysis

Statistical analysis was performed using IBM SPSS Statistics for Windows, Version 22.0 (IBM Corp., released 2013, Armonk, NY, USA). Continuous data are expressed as mean ± standard deviation and categorical variables are shown as percentages. The independent t-test or Mann–Whitney U test (if data were abnormally distributed) was used to compare continuous variables between groups. Categorical variables were analyzed using the Pearson chi-square test or Fisher exact test. Survival rates were analyzed with the Kaplan–Meier method. The mean patient and graft survivals in different groups were compared using the log-rank test. Cox regression analysis was used to calculate the multivariate hazard ratio (HR) and 95% confidence intervals (95% CI). Variables with a *p*-value <0.2 from the univariate analysis were included in the multivariate regression analysis. A two-sided *p*-value of less than 0.05 was considered statistically significant.

## 3. Results

### 3.1. Etiology of Liver Cirrhosis, Major EDC, and Comparison of No-maEDC and maEDC Groups

Chronic hepatitis infection caused cirrhosis in 52.6% of patients (14% hepatitis B [HBV] and 38.6% hepatitis C [HCV]) (Table 1). Of the 264 recipients analyzed, 66 (25%) received grafts with no EDC, 62 (23.5%) received grafts with miEDC, 125 (47.3%) received grafts with one maEDC, and 11 received grafts with two maEDC (4.2%). No patient received a graft with three maEDC (Figure 1). Seven patients (2.7%) were transplanted with steatotic grafts (BPS > 40%), 102 (38.6%) received grafts from donors older than 65 years, and 16 (6.1%) were transplanted with grafts with a CIT of >14 h. Five patients (1.9%) were transplanted with grafts from donors older than 65 years with a CIT of >14 h, five (1.9%) received steatotic grafts from donors older than 65 years, and two patients (0.8%) received steatotic organs with a CIT of >14 h. The multivariate analysis of the hazard ratios of EDC on one-year graft failure showed that a donor age of >65 years (HR 1.8, 95% CI 1.0–3.4, *p* = 0.049) and CIT of >14 h (HR 2.9, 95% CI 1.3–6.5, *p* = 0.008) had a significant impact on one-year graft failure. A BPS of >40% did not affect one-year graft failure (HR 1.8, 95% CI 0.6–5.1, *p* = 0.279). The mean donor age in the no-maEDC group was lower than the mean donor age in the maEDC group (49.82 ± 12.5 vs. 70.62 ± 12.46 years; *p* < 0.001). The mean CIT was also significantly shorter in the no-maEDC group (9.73 ± 2.19 vs. 10.81 ± 2.79 h; *p* < 0.001). There were no differences in donor gender, donor body mass index, and intensive care unit stay between the two groups. Also, the recipients of no-maEDC and maEDC grafts did not differ in age, gender, body mass index, and labMELD scores (Table 1).

Major EDC and no-maEDC grafts were distributed equally between recipients with different etiologies of cirrhosis (Table 1). Three (2.9%) of the 102 patients with HCV-related cirrhosis were retransplanted and six patients (5.9%) died because of disease recurrence during follow-up. Forty candidates (39.2%) with HCV-related cirrhosis received organs from donors older than 65 years. In these recipients, HCV recurrence caused graft failure in three patients (7.5%) and was the cause of death in one patient (2.5%) in the first year after transplantation. One-year graft and patient survival did not differ between recipients with HCV-related cirrhosis and non-HCV-related cirrhosis (*p* = 0.342 and *p* = 0.313, respectively), but there was a significant difference in five-year graft survival between these groups (*p* = 0.045). One-year and five-year graft and patient survival did not differ between recipients with HCV-related cirrhosis who were transplanted with livers from donors older than 65 years and those who received grafts from younger donors (*p* = ns for all categories). Also, we observed no difference in one-year and five-year graft and patient survival between recipients with HCV-related cirrhosis who were transplanted with grafts from donors older than 65 years before and after the introduction of the direct antiviral agents (DAA) in 2014 (*p* = ns for all categories). However, one-year and five-year graft survival were 100% in recipients with HCV-related cirrhosis who were transplanted with grafts from donors older than 65 years after 2014. Similarly, one-year patient survival was 100% in recipients with HCV-related cirrhosis who were transplanted after 2014 but declined to 67% at five years after transplantation.

### 3.2. Outcome Following Liver Transplantation for HCC

The one-year graft survival rate was 82.1% and the one-year retransplant rate was 12.1%. The 90-day, 1-year, 3-year, and 5-year patient survival rates were 87.9%, 80.6%, 70.8%, and 69%, and they increased to 100%, 92.9%, 88%, and 87.2% after censoring for mortality secondary to reasons other than graft failure-related complications. There were no differences in the causes of death between the recipients of no-maEDC and maEDC grafts (*p* = ns for all categories). Major morbidity (Clavien–Dindo ≥ IIIb) occurred in 35.2% of cases, but the rate of major morbidity did not differ significantly between recipients of no-maEDC and maEDC grafts (40.4% vs. 45.6%; *p* = 0.068) (Table 1).

### 3.3. Graft Survival and Major EDC

One-year graft survival was higher in recipients of no-maEDC grafts (Table 1). Early allograft dysfunction, primary nonfunction, and 30-day failure rates did not differ significantly between no-maEDC, miEDC, one maEDC, and two maEDC groups; however, 90-day graft failure was higher in recipients of organs with two maEDC (Table 2). One-year retransplantation rates did not differ between recipients of steatotic grafts (BPS > 40%) and recipients of no-maEDC organs (*p* = 0.085). One-year retransplantation rates were higher in recipients of grafts from donors older than 65 years (14.7% vs. 6.3%) and with a CIT of >14 h (25% vs. 6.3%) (*p* = 0.045 and 0.030, respectively). The combination of donor age >65 years and CIT >14 h yielded a 40% one-year retransplantation rate. Compared with no-maEDC liver transplantation, BPS of >40% alone, or combined with a donor age of >65 years or CIT of >14 h did not affect the retransplantation rates (*p* = ns for all combinations).

### 3.4. Patient Survival and Major EDC

**Figure 2:**
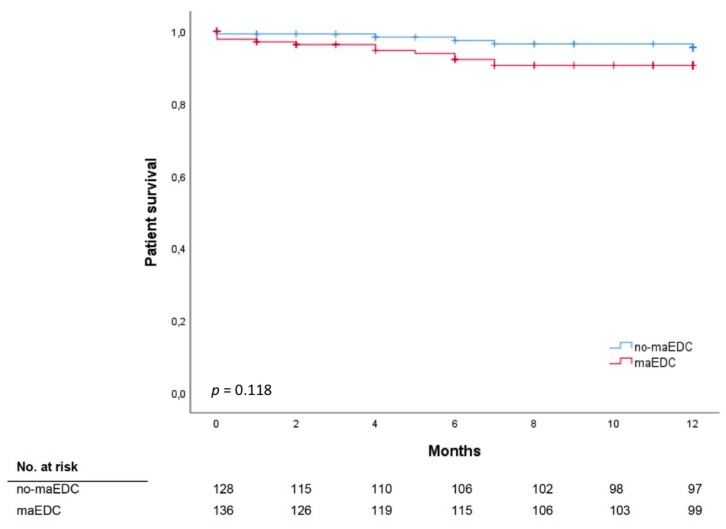
Patient survival does not significantly differ between recipients of no-maEDC and of maEDC grafts after censoring for mortality secondary to reasons other than graft failure-related complications (log-rank *p* = 0.118).

One-year patient survival did not differ between recipients of no-maEDC and maEDC grafts (82.4% vs. 78.9%; *p* = 0.555), and still did not differ after censoring for mortality secondary to reasons other than graft failure-related complications (95.5% vs. 90.5%; *p* = 0.118) (Table 1, Figure 2). Likewise, 30-day, 90-day, and 1-year mortality rates did not differ between recipients of no-maEDC, miEDC, one maEDC, and two maEDC grafts (Table 3 and Table 4).

### 3.5. Univariate and Multivariate Analyses

Cox regression univariate and multivariate analyses identified labMELD scores and maEDC to be independently associated with one-year graft survival (HR 0.915, 95% CI 0.846–0.991, *p* = 0.029; HR 2.603, 95% CI 1.340–5.055, *p* = 0.005, respectively) (Table 5). Cox regression univariate and multivariate analyses identified female recipient gender to be independently associated with one-year patient mortality after censoring for mortality secondary to reasons other than graft failure-related complications (HR 3.671, 95% CI 1.4–13.231, *p* = 0.011) (Table 6). The analysis revealed no association between maEDC grafts and one-year patient mortality (HR 2.266, 95% CI 0.798–6.434, *p* = 0.124).

### 3.6. MELD Score and Major EDC

**Figure 3:**
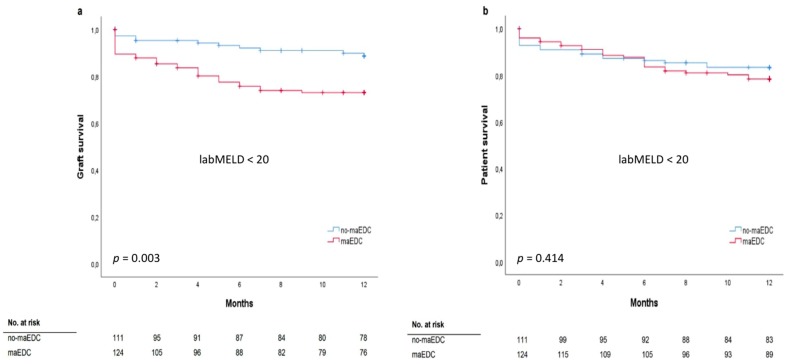
Graft survival (**a**) (log-rank *p* = 0.003) and patient survival analysis (**b**) (log-rank *p* = 0.414) of recipients with a labMELD score of <20 who were transplanted with no-maEDC and maEDC grafts.

**Figure 4:**
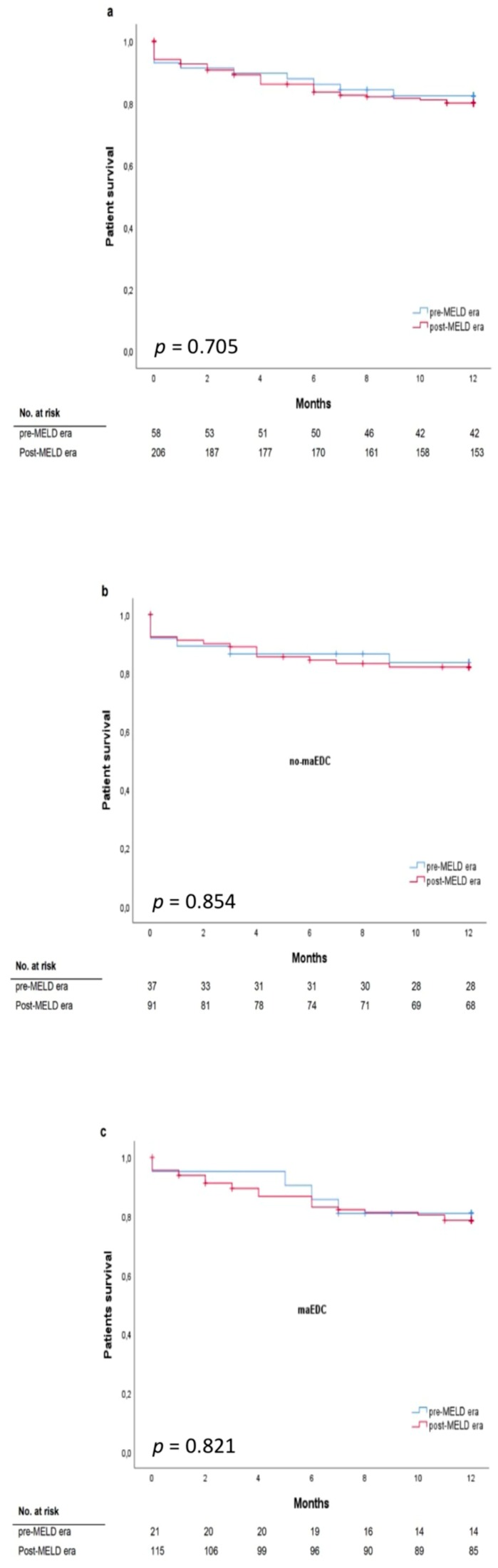
Patient survival does not significantly differ before and after establishment of model of end-stage liver disease (MELD) criteria in all patients (**a**), patients without maEDC (**b**), and patients with maEDC (**c**) (log-rank *p* = 0.705, 0.854, and 0.821).

The mean labMELD and eMELD scores were 12.45 ± 6.19 and 27.24 ± 4.23, respectively. The labMELD or the eMELD score was used for allocation purposes and the higher score according to the waiting list was chosen as the matchMELD score (23.20 ± 8.41). A total of 205 liver transplants (78%) were performed after the MELD score-based allocation was introduced in December 2006. In 176 cases (66.7%), liver grafts were allocated according to the eMELD score. Graft survival differed between the recipients with a labMELD score of <20 who received no-maEDC and those who received maEDC grafts (log-rank *p* = 0.003) (Figure 3a). Graft survival was not significantly different in recipients with a labMELD score of ≥20 who were transplanted with no-maEDC grafts or those transplanted with maEDC grafts (log-rank *p* = 0.404). No differences were observed in patient survival after correcting for a labMELD score with a cut-off value of 20 (labMELD score < 20, log-rank *p* = 0.414) (Figure 3b) and a labMELD score of ≥20 (log-rank *p* = 0.669). Also, after censoring for death with functioning graft, no differences were observed in patient survival after correcting for a labMELD score with a cut-off value of 20 (labMELD < 20, log-rank *p* = 0.139; labMELD ≥ 20, log-rank *p* = 0.401). Patient survival did not differ before and after the MELD score-based allocation system was introduced (log-rank, *p* = 0.705) (Figure 4a). Likewise, MELD score-based allocation did not affect patient survival in both recipients of no-maEDC and maEDC grafts (log-rank, *p* = 0.854 and *p* = 0.821, respectively) (Figure 4b,c). The average time on the waiting list of the collective was 375 days. The time on the waiting list did not differ between recipients of maEDC grafts and recipients of no-maEDC organs (Table 1).

### 3.7. Milan Criteria and Major EDC

**Figure 5:**
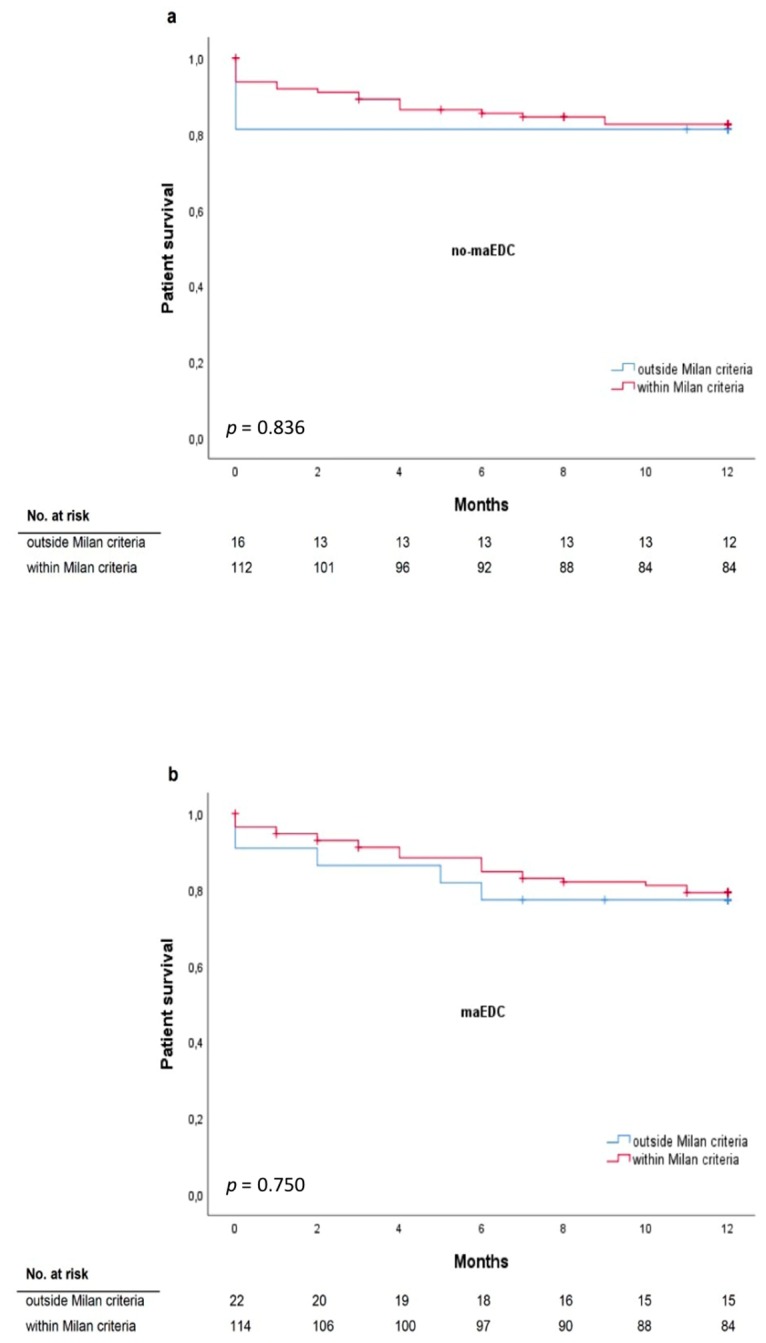
One-year survival for patients who met or did not meet the Milan criteria* transplanted with grafts without maEDC (**a**) (log-rank *p* = 0.836) and with maEDC (**b**) (log-rank *p* = 0.750). * All patients met the University of California San Francisco (UCSF) criteria

Milan criteria were met by 85.6% of the recipients and all the recipients met the UCSF criteria. The median alpha-fetoprotein level was 13 IU/mL and tumor differentiation was assessed as G1 in 23.5% of patients, G2 in 65.5% of patients, and G3 in 11% of patients. Before liver transplantation, 87.5% of recipients underwent bridging treatment. One-year patient survival did not differ between recipients who met the Milan criteria and those who did not (77.8% and 76.2%; *p* = 0.781). Also, one-year patient survival did not differ between recipients who did and did not meet the Milan criteria and who received grafts with and without maEDC (log-rank *p* = 0.836 and *p* = 0.750, respectively) (Figure 5). One-year, three-year, and five-year recurrence rates were 23.5%, 34.8%, and 40.9%, respectively. HCC recurrence rates did not differ between recipients of maEDC and no-maEDC grafts (8.6% vs. 11.8%, *p* = 0.395). The five-year recurrence-free survival did not differ between the maEDC and no-maEDC groups (*p* = 0.113).

## 4. Discussion

The number of patients waiting for a liver transplantation exceeds the number of available organs in most countries [3]. Liver transplantation is the only promising treatment because it involves radical oncological resection and improves the underlying liver dysfunction; however, patients with HCC have the longest waiting times for a transplant and 7–55% of them drop out of the waiting list because of disease progression [2,13,14,15]. The prevalence of HCC is similar in the USA and Europe, and according to the United Network for Organ Sharing and European Liver Transplant Registry reports, so were the transplant rates (17.42% and 17.62%, respectively) for HCC between 2002 and 2016 in the two regions [16,17,18]. Unlike the USA, Germany has an opt-in system, which means that people have to actively sign up to be considered as donors after death. Also, the rates of living donation liver transplantation in Germany and other Eurotransplant countries are undulating [16,19]. The consequent chronic organ shortage has increased the transplantation of EDC grafts in Eurotransplant countries whose suboptimal quality has worsened outcomes after liver transplantation [3]. Therefore, despite organ shortage, these grafts are often discarded because they are considered unsuitable for transplantation [4]. We were able to show that transplantation of grafts with more than one maEDC into patients with labMELD scores ≥20 yielded worse outcomes and we therefore suggested that the number of maEDC should be balanced with the recipient’s condition [3]. The results of the present study show that patients with liver cirrhosis and HCC who generally are in a better condition may be able to overcome the negative impact of maEDC and could be transplanted with maEDC grafts without impairing patient survival. The survival rates in the study are comparable with those of other European and Asian centers, but are lower than the rates reported in the USA, which can be explained by the lower quality of liver grafts available for transplantation in Eurotransplant [16,17,20,21,22,23].

### 4.1. Major EDC and Clinical Outcome After Liver Transplantation

Macrovesicular liver steatosis is an essential determinant of graft function; primary nonfunction rates are up to 80% in cases of severe steatosis and between 1.4% and 8.5% when fatty livers are excluded [24,25]. Mild steatosis (<30%) does not affect long-term graft function or patient survival, whereas most transplant surgeons would discard grafts with severe steatosis (>60%). Using moderately steatotic grafts (30–60%) for transplantation remains controversial [25,26,27]. These grafts qualify as marginal because they have been associated with poor clinical outcomes, especially when combined with prolonged CIT or advanced donor age, and must be therefore carefully matched with appropriate recipients [3]. In the current study, a BPS of >40% did not affect graft or patient survival in recipients with HCC and liver cirrhosis who were in good clinical condition and had labMELD scores <20, confirming that maEDC grafts may be suitable for these recipients but should not be considered for transplant candidates with higher labMELD scores [3]. The simultaneous presence of BPS and longer CIT (>14 h) or advanced donor age (>65 years) yielded no difference in retransplant rates compared with the no-maEDC transplant cases. This could be partially explained by the low number of liver transplantations with combined maEDC, but in the majority of cases, grafts were allocated according to the HCC eMELD score, which reflects the oncologic status, unlike the labMELD score, which reflects the severity of underlying liver disease and function. The discrepancy between the labMELD and eMELD scores suggests that recipients in better clinical condition with lower labMELD scores and higher reserves (such as HCC patients with cirrhosis) might experience less injury from graft steatosis and reduce the risk of poor clinical outcome caused by moderate BPS. Also, moderate BPS did not affect the HCC recurrence rates.

The mean age of liver donors is increasing worldwide, despite evidence that advanced donor age impacts early mortality after transplantation, mainly because of increased cellular senescence and reduced regeneration of hepatocytes [3,28,29]. The liver parenchyma is more vulnerable to ischemia-reperfusion injury and inflammatory cytokine responses before and after transplantation [29,30]. The risk of graft loss increases linearly from a donor age of 25 up to 80 years old, but substantial survival benefits have also been reported [21,31]. Our results and retransplant rates are in line with these reports. Unlike in the USA, transplanting grafts from older donors is common practice in Eurotransplant because of the chronic organ donor shortage [16,32]. The median donor age increased from 43 to 55 years in only 15 years within Eurotransplant countries [21]. In the present study, a donor age of >65 years doubled the risk of one-year graft failure but did not increase the risk of one-year patient mortality. This is in agreement with previous reports that grafts from older donors should be preferred for recipients with low labMELD scores [33]. Defining the effect of increasing donor age is very important, especially in HCV-infected patients, because advanced donor age accelerates fibrosis progression in patients with recurring HCV and decreases graft and patient survival [29]. Therefore, grafts from older donors should not be allocated to HCV-infected candidates [33,34]. The number of transplant candidates with HCV-induced cirrhosis at our center peaked in 2011 and declined steadily until 2018. The interaction between advanced donor age and HCV recurrence may have been eradicated by DAA that cure HCV either before or after transplantation [29,35]. Although DAA medications were not available during most of the study period, HCV recurrence caused graft failure in only three patients and death in only one patient in the first year after transplantation. One-year graft and patient survival did not differ between the recipients with HCV-related liver cirrhosis and non-HCV-related cirrhosis, but five-year graft survival was higher in non-HCV liver graft recipients, which is in agreement with other reports [16,36]. Interestingly, graft and patient survival did not differ between recipients with HCV-related cirrhosis who were transplanted with livers from donors older than 65 years and those who were transplanted with grafts from younger donors. Also, we observed no significant difference in one-year and five-year graft and patient survival between recipients with HCV-related cirrhosis who were transplanted with grafts from donors older than 65 years before and after the introduction of DAA in 2014. However, there was no graft failure during the first five years following transplantation after 2014. Similarly, no patient death was observed during the first year following transplantation in recipients with HCV-related cirrhosis who were transplanted with grafts from donors older than 65 years after 2014. This observation might be due to the low numbers of HCV-related cirrhosis transplant cases after DAA were introduced, but also suggests that older livers may be an option for patients with treated HCV. Donor age had no effect on the HCC recurrence rates.

A CIT of >14 h is a maEDC associated with an increased risk of organ failure and early HCC recurrence [3]. Nagai et al. reported twofold increased risk of hazard for CIT longer than 10 h and median time to HCC recurrence of 0.9 years [37]. After an extended CIT, graft outcome depends on the ability of the transplanted liver to recover from ischemia, which might be difficult in grafts from older donors [28,29,30,38]. Consequently, organs with longer CIT are often discarded because they are considered unsuitable for transplantation. In the current study, CIT > 14 h increased the risk of one-year graft failure threefold but did not affect one-year patient survival. A CIT > 14 h was not associated with increased HCC recurrence, but it yielded the highest one-year retransplant rate—analyzed alone and in combination with advanced donor age.

### 4.2. Major EDC and the MELD Score

Transplanting grafts with more than one maEDC into recipients with labMELD scores ≥20 worsens the outcome [3]. Major morbidity rates were comparable in HCC patients with cirrhosis, who received maEDC and no-maEDC grafts, and transplanting grafts with ≥1 maEDC bore no risk for early allograft dysfunction, primary nonfunction, or 30-day graft failure in these recipients. The morbidity and retransplant rates are comparable with those already reported [39,40,41]. Grafts with ≥1 maEDC were associated with a reduced short-term graft survival, but they did not affect one-year patient survival and one-year patient survival after censoring for death with functioning graft. This was confirmed by our univariate and multivariate analyses of graft survival. Considering the organ donor shortage and the similar one-year mortality rates between the groups, maEDC grafts that would otherwise be discarded may be acceptable for patients with HCC and cirrhosis, who would otherwise die from disease progression while waiting for a transplant. Although no differences were observed in graft or patient survival after liver transplantation between 2001–2006 (pre-MELD era in the Eurotransplant region) and 2007–2018 (MELD era), improvements in clinical care during these 18 years probably influenced the outcomes following liver transplantation.

Very short (<6 months) or long (>18 months) waiting times from HCC diagnosis to liver transplantation have been associated with a 60% increased risk of HCC recurrence compared to those with a waiting time of 6–18 months. The waiting time “sweet spot” of 6–18 months should therefore be targeted to minimize HCC recurrence [42]. The time on the waiting list in the current study was comparable with previous reports [42,43]. Transplantation of maEDC grafts did not reduce the time on the waiting list, which can be explained by the lack of systematic allocation of maEDC grafts to transplant candidates with HCC, and by the lack of informed consent from all patients for transplantation with EDC livers.

### 4.3. Milan and UCSF Criteria and Risk Factors for Increased Mortality after Liver Transplantation

The Milan criteria are a conventional selection tool for patients with HCC and were incorporated in the Barcelona Clinic Liver Cancer and the United Network for Organ Sharing (UNOS) staging systems [44]. Extending the Milan criteria may worsen the survival after transplantation [45]. In the current study, one-year patient mortality did not differ between patients who met the Milan criteria and those who did not. Also, patient survival did not differ between recipients who did and did not meet the Milan criteria and who received grafts with and without maEDC. Furthermore, maEDC neither increased the HCC recurrence rates nor did they affect the five-year recurrence-free survival. Similar to our previous findings, univariate and multivariate analyses indicated that female recipients have an increased risk of one-year postoperative mortality [11]. Decreased chances of survival were reported when female livers were transplanted into male recipients, and estrogens may affect the long-term postoperative outcome [46,47]. In contrast to these findings, our results indicated that female recipient gender influenced the one-year patient mortality. Similar to our previous study, we were not able to explain the findings observed in the multivariate analysis [11].

Limitations of the present study are the retrospective single-center study design and the modest number of transplants analyzed. The number of recipients who received steatotic grafts with a BPS of >40% was low; therefore this subgroup may not be representative. When discussing potential adverse effects of maEDC, predefined thresholds must also be considered. Reese et al. showed that a donor age of ≥45 years and a CIT of ≥12 h negatively affected the 90-day graft outcome, and this negative effect may also occur with a low ischemia time of 9 h or a younger donor age of 40 years. Furthermore, the ‘number needed to harm’ of 16 patients indicated that, for every 16 livers from donors of ≥45 years old subjected to a cold ischemia of ≥12 h, one more graft would fail compared with allografts from donors aged ≥45 years subjected to a ischemia time of less than 12 h [48].

## 5. Conclusions

In conclusion, maEDC grafts may provide an alternative for patients with HCC and liver cirrhosis who are waiting for a life-saving transplant. However, whether maEDC organs can be allocated to transplant candidates with HCC and liver cirrhosis needs to be considered with great caution; such decisions cannot be made easily. This study is a risk assessment based on retrospective data analysis, and no score can replace the experience and expertise of the transplant surgeons. Furthermore, such score must be based on the results of prospective, randomized, and strictly controlled trials. Based on the transplant experience in Germany and Eurotransplant, where the donor pool is very limited, the maximal utilization of EDC grafts is of extreme importance and measures need to be taken to increase the volume of the pool. This study takes steps to achieve this because, compared with living donor liver transplantation, maEDC-transplantation is more suitable since a large-volume transplant center and vast experience with living donation transplantation are not required. Without impairing the patient survival or increasing the tumor recurrence rates, transplanting liver grafts with maEDC may be a reasonable option for patients with HCC and liver cirrhosis.

## Figures and Tables

**Table 1:** Demographic characteristics, clinical parameters of the donor and recipient collective, etiology of liver cirrhosis, and comparison of no major extended donor criteria (maEDC) organ recipients and recipients of grafts with ≥1 maEDC.

	Total *n* = 264	maEDC = 0 *n* = 128	maEDC ≥ 1 *n* = 136	*p*
**Donor, *n* = 264**				
BPS > 40%	14 (5.3%)	0% 50 ± 13	10.3% 71 ± 12	<0.001 <0.001
Age (years, mean ± SD)	61 ± 16			
>65 years	59 (39.6%)			
CIT (hours, mean ± SD)	10.2 ± 2.53	9.7 ± 2.2	10.8 ± 2.8	<0.001 0.303
>14 h	15 (10.1%)			
Gender				
Male	58%	61%	55%	
Female	42%	39%	45%	
BMI (kg/m^2^, mean ± SD)	26.4 ± 3.7	26.1 ± 3.6	26.7 ± 3.8	0.196
ICU/MV (days, mean ± SD)	4.2 ± 4.1	4.4 ± 4	4.0 ± 4.1	0.436
**Recipient, *n* = 264**				
Age (years, mean ± SD)	57 ± 7	57 ± 7	56 ± 7	0.318
Gender				0.84
Male	86%	85%	86%	
Female	14%	15%	14%	
BMI (kg/m^2^, mean ± SD)	27.4 ± 4.3	27.7 ± 4.1	27.1 ± 4.4	0.201
labMELD score (mean ± SD)	12.45 ± 6.2	12.75 ± 6.8	12.2 ± 5.5	0.444 0.247
<20	89%	87%	91%	
≥20	11%	13%	9%	
eMELD	27.24 ± 4.2	28.4 ± 4.8	26.4 ± 3.6	0.004
MatchMELD	23.2 ± 8.4	22.4 ± 9.7	23.9 ± 6.9	0.137
Recipients meeting Milan criteria *	85.6%	87.5%	83.8%	0.395
Time on the waiting list (days ± SD)	375 ± 528	314 ± 303	435 ± 678	0.062
Major morbidity (Clavien–Dindo cl. ≥ IIIb)	35.2%	40.4%	45.6%	0.068
1-year graft survival	82.1%	89.5%	75.3%	0.003
1-year patient survival (liver failure ass.)	92.9%	95.5%	90.5%	0.118
1-year patient mortality	18.9%	17.2%	20.6%	0.531
**Etiology of liver cirrhosis**				
Hepatitis	139 (52.6%)	70 (54.7%)	69 (50.7%)	0.539
HBV-induced cirrhosis	37 (14%)	18 (14.1%)	19 (14%)	
HCV-induced cirrhosis	102 (38.6%)	52 (40.6%)	50 (36.8%)	
Alcohol-induced cirrhosis	90 (34.1%)	45 (35.2%)	45 (33.1%)	0.795
Cryptogenic liver cirrhosis	22 (8.3%)	10 (7.8%)	12 (8.8%)	0.826
Autoimmune hepatitis	5 (1.9%)	1 (0.8%)	4 (2.9%)	0.371
Nonalcoholic steatohepatitis	3 (1.1%)	1 (0.8%)	2 (1.5%)	0.999
Hemochromatosis	2 (0.8%)	1 (0.8%)	1 (0.7%)	0.999
Biliary atresia	1 (0.4%)	0 (0%)	1 (0.7%)	0.999
Inflammatory adenoma HCC	1 (0.4%)	0 (0%)	1 (0.7%)	0.999
Primary biliary cholangitis	1 (0.4%)	0 (0%)	1 (0.7%)	0.999

Abbreviations: EDC, extended donor criteria; maEDC, major EDC; SD, standard deviation; BPS, biopsy-proven macrovesicular steatosis; CIT, cold ischemia time; BMI, body mass index; ICU, duration of the intensive care unit stay before organ procurement; MV, duration of mechanical ventilation of the donor before organ procurement; labMELD, laboratory model for end-stage liver disease; eMELD, exceptional MELD; HBV, hepatitis B virus; HCV, hepatitis C virus; HCC, hepatocellular carcinoma. * Underlying diseases other than hepatitis and alcohol were set as baseline.

**Table 2:** Graft loss following liver transplantation in patients with hepatocellular carcinoma (HCC).

	no-EDC (*n* = 66)	miEDC (*n* = 62)	maEDC = 1 (*n* = 125)	maEDC = 2 (*n* = 11)	*p*
EAD, n (%)	29 (43.9)	37 (59.7)	61 (48.8)	7 (63.6)	0.249
PNF, n (%)	1 (1.5)	1 (1.6)	9 (7.2)	1 (9.1)	0.159
30-day graft loss, n (%)	1 (1.5)	3 (4.8)	11 (8.8)	2 (18.2)	0.086
90-day graft loss, n (%)	2 (3.0)	4 (6.5)	15 (12.0)	3 (27.3)	0.028

EAD, early allograft dysfunction; PNF, primary nonfunction; EDC, extended donor criteria; miEDC, minor EDC; maEDC, major EDC (BPS > 40%, CIT > 14 h, donor age > 65 years).

**Table 3:** Patient mortality following liver transplantation in patients with HCC.

	no-EDC (*n* = 66)	miEDC (*n* = 62)	maEDC = 1 (*n* = 125)	maEDC = 2 (*n* = 11)	*p*
30-day mortality, n (%)	6 (9.1)	4 (6.5)	5 (4.0)	1 (9.1)	0.534
90-day mortality, n (%)	8 (12.1)	5 (8.1)	10 (8.0)	1 (9.1)	0.803
1-year mortality, n (%)	13 (19.7)	9 (14.5)	25 (20.0)	3 (27.3)	0.705

EAD, early allograft dysfunction; PNF, primary nonfunction; EDC, extended donor criteria; miEDC, minor EDC; maEDC, major EDC (BPS > 40%, CIT > 14 h, donor age > 65 years).

**Table 4:** Patient mortality following liver transplantation in patients with HCC after censoring for mortality secondary to reasons other than graft failure-related complications.

	no-EDC (*n* = 66)	miEDC (*n* = 62)	maEDC = 1 (*n* = 125)	maEDC = 2 (*n* = 11)	*p*
30-day mortality, n (%)	0 (0)	1 (1.6)	2 (1.6)	1 (9.1)	0.154
90-day mortality, n (%)	0 (0)	1 (1.6)	4 (3.2)	1 (9.1)	0.217
1-year mortality, n (%)	4 (6.1)	1 (1.6)	11 (8.8)	1 (9.1)	0.296

EAD, early allograft dysfunction; PNF, primary nonfunction; EDC, extended donor criteria; miEDC, minor EDC; maEDC, major EDC (BPS > 40%, CIT > 14 h, donor age > 65 years).

**Table 5:** Univariate and multivariate cox regression analyses of factors associated with 1-year graft survival.

	Univariate Analysis			Multivariate Analysis		
	HR	95% CI	*p*	HR	95% CI	*p*
Recipient age	1.001	0.959–1.044	0.971			
Recipient female gender	1.587	0.763–3.301	0.217			
maEDC	2.605	1.342–5.059	0.005	2.603	1.340–5.055	0.005
HCC grade G3	1.075	0.424–2.727	0.879			
Recipient BMI > 30 kg/m^2^	0.780	0.373–1.630	0.509			
labMELD	0.919	0.851–0.991	0.029	0.915	0.846–0.991	0.029
Underlying disease (cause of LC)						
Other *	baseline	baseline	baseline			
Hepatitis	0.704	0.237–2.091	0.527			
Alcohol	0.875	0.468–1.639	0.677			
Recipients meeting Milan criteria	1.580	0.759–3.287	0.221			
LC Child-Pugh score (B/C vs. A)	0.783	0.429–1.428	0.425			

EDC, extended donor criteria; maEDC, major extended donor criteria; HR, hazard ratio; CI, confidence interval; BMI, body mass index; labMELD, laboratory model for end-stage liver disease; LC, liver cirrhosis; HCC, hepatocellular carcinoma. * Underlying diseases other than hepatitis and alcohol were set as baseline.

**Table 6:** Univariate and multivariate cox regression analyses of factors associated with 1-year patient survival.

	Univariate Analysis			Multivariate Analysis		
	HR	95% CI	*p*	HR	95% CI	*p*
Recipient age	0.992	0.926–1.064	0.827			
Recipient female gender	3.267	1.208–8.836	0.02	3.671	1.4–13.231	0.011
maEDC	2.266	0.798–6.434	0.124	2.556	0.872–8.281	0.081
HCC grade G3	1.031	0.236–4.507	0.968			
Recipient BMI > 30 kg/m^2^	0.876	0.286–2.686	0.817			
labMELD	0.92	0.814–1.038	0.176	0.916	0.813–1.031	0.145
Underlying disease (cause of LC)						
Other *	baseline	baseline	baseline			
Hepatitis	1.563	0.192–12.706	0.654			
Alcohol	3.027	0.383–23.893	0.293			
Recipients meeting Milan criteria	1.922	0.627–5.895	0.253			
LC Child-Pugh score (B/C vs. A)	0.618	0.228–1.671	0.343			

EDC, extended donor criteria; maEDC, major extended donor criteria; HR, hazard ratio; CI, confidence interval; BMI, body mass index; labMELD, laboratory model for end-stage liver disease; LC, liver cirrhosis; HCC, hepatocellular carcinoma. * Underlying diseases other than hepatitis and alcohol were set as baseline.
